# Rational approximation of golden angles: Accelerated reconstructions for radial MRI

**DOI:** 10.1002/mrm.30247

**Published:** 2024-09-09

**Authors:** Nick Scholand, Philip Schaten, Christina Graf, Daniel Mackner, H. Christian M. Holme, Moritz Blumenthal, Andrew Mao, Jakob Assländer, Martin Uecker

**Affiliations:** 1 Institute of Biomedical Imaging, Graz University of Technology, Graz, Austria; 2 Center for Biomedical Imaging, Department of Radiology, NYU School of Medicine, New York, New York, USA; 3 German Centre for Cardiovascular Research (DZHK), partner site Lower Saxony, Göttingen, Germany; 4 Department of Pediatrics, The University of British Columbia, Vancouver, British Columbia, Canada; 5 Department of Physics and Astronomy, The University of British Columbia, Vancouver, British Columbia, Canada; 6 Institute for Diagnostic and Interventional Radiology, University Medical Center Göttingen, Göttingen, Germany; 7 Center for Advanced Imaging Innovation and Research (CAI2R), Department of Radiology, New York University School of Medicine, New York, New York, USA; 8 Vilcek Institute of Graduate Biomedical Sciences, New York University School of Medicine, New York, New York, USA; 9 BioTechMed-Graz, Graz, Austria; 10 Cluster of Excellence “Multiscale Bioimaging: from Molecular Machines to Networks of Excitable Cells” (MBExC), University of Göttingen, Göttingen, Germany

**Keywords:** dynamic MRI, golden angle, golden ratio sampling, radial sampling, rational approximation

## Abstract

**Purpose:**

To develop a generic radial sampling scheme that combines the advantages of golden ratio sampling with simplicity of equidistant angular patterns. The irrational angle between consecutive spokes in golden ratio-based sampling schemes enables a flexible retrospective choice of temporal resolution, while preserving good coverage of k-space for each individual bin. Nevertheless, irrational increments prohibit precomputation of the point-spread function (PSF), can lead to numerical problems, and require more complex processing steps. To avoid these problems, a new sampling scheme based on a rational approximation of golden angles (RAGA) is developed.

**Methods:**

The theoretical properties of RAGA sampling are mathematically derived. Sidelobe-to-peak ratios (SPR) are numerically computed and compared to the corresponding golden ratio sampling schemes. The sampling scheme is implemented in the BART toolbox and in a radial gradient-echo sequence. Feasibility is shown for quantitative imaging in a phantom and a cardiac scan of a healthy volunteer.

**Results:**

RAGA sampling can accurately approximate golden ratio sampling and has almost identical PSF and SPR. In contrast to golden ratio sampling, each frame can be reconstructed with the same equidistant trajectory using different sampling masks, and the angle of each acquired spoke can be encoded as a small index, which simplifies processing of the acquired data.

**Conclusion:**

RAGA sampling provides the advantages of golden ratio sampling while simplifying data processing, rendering it a valuable tool for dynamic and quantitative MRI.

## 1 INTRODUCTION

Radial trajectories were the first sampling schemes used in MRI^1^ and are now widely used in dynamic imaging,^2–4^ compressed sensing,^5–7^ and quantitative MRI.^8–12^ Radial trajectories have several advantages: They are ideally suited for continuous acquisitions,^13^ are robust to motion,^2,14^ and they repeatedly acquire the k-space center which can be used for correction of gradient imperfections,^15,16^ self-navigation,^17^ and calibration of coil sensitivities.^18^ For these reasons, radial acquisitions are increasingly used in clinical applications including imaging of the heart, breast, abdomen, and brain.^19^ Nevertheless, the reconstruction of data acquired with non-Cartesian trajectories has a high computational cost, especially for iterative reconstruction algorithms. To reduce computation time, reconstruction techniques often use preprocessing steps including prior interpolation/shifting of the data onto a Cartesian grid^20–22^ or replacing the joint operation of interpolation and gridding by a convolution with the point-spread function (PSF) in Toeplitz-based methods.^23–25^

Radial sampling schemes differ in the angle between spokes and in the overall temporal ordering of the acquisition of the spokes. One of the simplest schemes is an equidistant angular pattern in which the spokes are homogeneously distributed over a whole circle or half circle. One advantage is that the acquired data is self-explanatory, that is, the angle between spokes can be derived from the total number of radial projections. Another advantage is that only a finite number of angular positions are sampled, which then allows sharing of precomputed information about quantities such as the PSF which may save memory or computation time.

While equidistant sampling is a good choice for static imaging, other sampling schemes are preferable for dynamic imaging. To improve spatiotemporal k-space coverage in the presence of motion or dynamic contrast changes turn-based acquisition schemes are used that reorder the spokes so that consecutive spokes are separated by larger angles.

A disadvantage of such schemes is that the temporal resolution is determined by the temporal footprint of a single turn. A more flexible solution is offered by golden ratio based sampling.^26,27^ By choosing an irrational angle between spokes based on the golden ratio, good k-space coverage is ensured simultaneously for different bin sizes. Temporal resolution can then be selected retrospectively. Therefore, golden ratio-based schemes are among the most common radial sampling schemes.^28^ Nevertheless, compared to equidistant angular sampling, irrational angles also have a disadvantage: Every spoke samples a unique projection and this prevents sharing of precomputed information such as the PSF between different temporal frames of an acquired time series.

This work develops a new radial sampling scheme that combines the advantages of equidistant angular patterns with the advantages of golden ratio sampling. The method, termed Rational Approximation of Golden Angles (RAGA), exploits a generalized Fibonacci formulation to compute a rational approximation of golden ratio-based angles. The concept of RAGA is introduced, and its theoretical properties are described. Numerically and experimentally, it is confirmed that RAGA sampling schemes closely match the corresponding irrational sampling schemes. Feasibility is demonstrated with phantom and in vivo scans.

## 2 | THEORY

### 2.1 | Rational approximation of golden angles

Golden ratio sampling as published by Winkelmann et al.^26^ is based on the number known as the golden ratio, that is,

(1) $$\tau \;:=\frac{\sqrt{5}\;+\;1}{2},$$

where the angle between consecutively acquired spokes is then given by

(2) $$\psi^{1}:=\frac{\pi }{\tau }\approx 111.245^{\circ }.$$

Wundrak et al.^27,29^ generalized this concept also to smaller angles by defining the N-th golden ratio angle as

(3) $$\psi^{N}:=\frac{\pi }{\tau \;+\;N\;-\;1}\;\text{and}\;N=1,\;2,\ldots \;.$$

For N>1, the $\psi^{N}$ are often called tiny golden angles. All these angles are defined with respect to π, that is, for a half-circle, and are irrational numbers multiplied by π. Note that $\psi^{1}$ is not the usual Golden Angle Φ≈137.508° which is defined as

(4) $$\Phi \;:=2\pi \left(1-\frac{1}{\tau }\right)=2\pi -2\psi^{1}.$$

The basic idea behind RAGA is to find a suitable rational approximation for the golden ratio angles $\psi^{N}$. Besides Equation (3), the golden ratio angle $\psi^{N}$ with index N can also be derived from the generalized Fibonacci series^30^

(5) $$G_{i}^{N}:=G_{i-1}^{N}+G_{i-2}^{N}\;\text{with}\;G_{1}^{N}:=1\;\text{and}\;G_{2}^{N}\;:=N.$$

The golden ratio angle $\psi^{N}$ is given by^27^

(6) $$\psi^{N}=\pi \cdot \underset{i\to \infty }{lim}\;\frac{G_{i-1}^{1}}{G_{i}^{N}}.$$

A rational approximation relative to π can be obtained by approximating $\psi^{N}$ with a finite order i as

(7) $$\psi^{N}\approx \psi_{i}^{N}\;:=\pi \cdot \frac{G_{i-1}^{1}}{G_{i}^{N}}.$$

The higher the order i the more accurate the approximation of $\psi^{N}$ becomes. An overview about different angles for various approximation orders i is shown in Table 1.

### 2.2 | RAGA sampling

#### 2.2.1 | Combining equidistant angular and golden ratio sampling

By using a rational approximation for the angle $\psi^{N}$ in Equation (7), the sampling scheme is fully defined in terms of the two integers $G_{i}^{N}$ and $G_{i-1}^{1}$. Here, $G_{i}^{N}$ defines a fundamental base angle

(8) $$\phi_{i}^{N}:=\frac{\pi }{G_{i}^{N}},$$

as an integer fraction of the half-circle and $G_{i-1}^{1}$ defines an index increment between consecutive spokes. Multiples of the base angle generate exactly the same angular positions that also occur in an equidistant angular sampling scheme with $S:=G_{i}^{N}$ spokes.

By choosing the increment between two temporally consecutive spokes as $G_{i-1}^{1}$, the angle between both spokes is

(9) $$\psi^{N}\approx G_{i-1}^{1}\cdot \phi_{i}^{N}.$$

**FIGURE 1:**
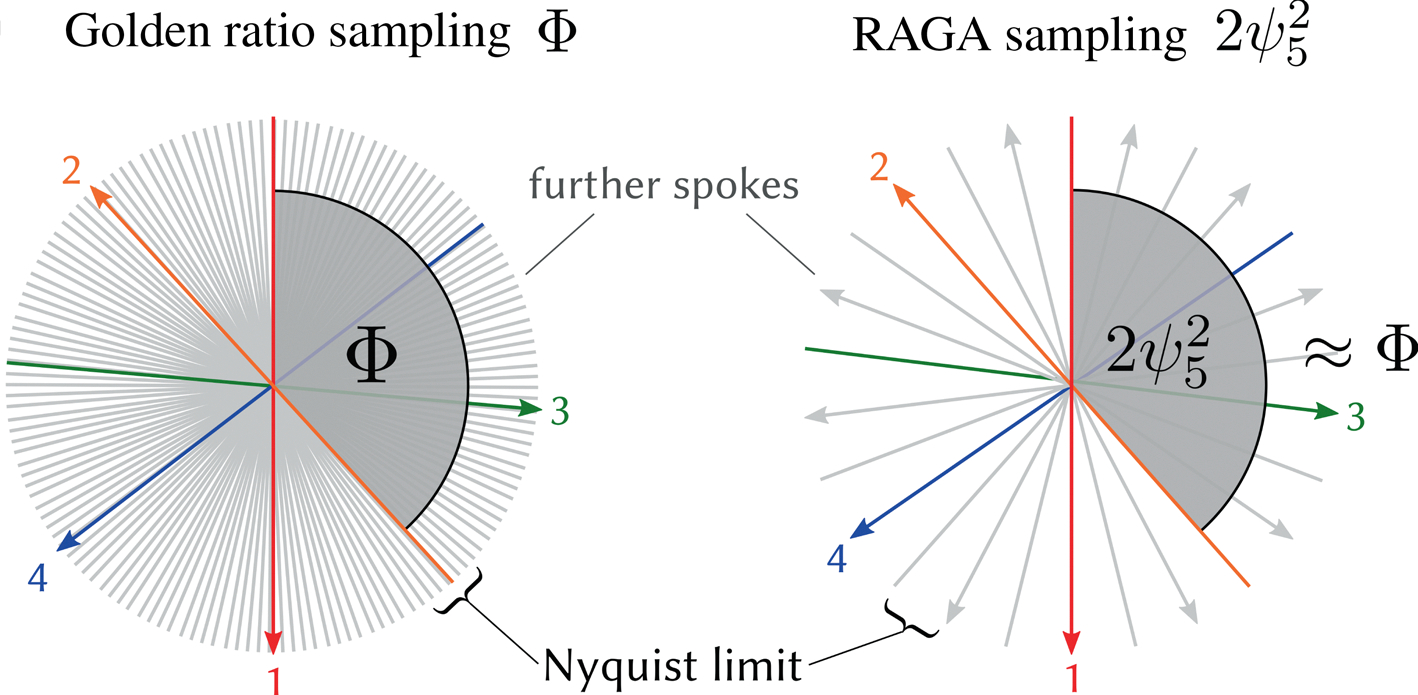
Comparison of a golden ratio (left) and a rational approximation of golden angles (RAGA) (right) sampling scheme. The golden ratio sampling scheme uses the golden angle Φ. The first four spokes of the trajectory are highlighted. Because the angle is irrational the spoke angles never repeat and new projections are acquired each time. The RAGA sampling scheme approximates Φ with an approximation order of i=5. It acquires the same set of spokes as an equidistant angular pattern with 13 spokes, but reordered so that the angle between temporally consecutive spokes approximates an angle of Φ. The pattern repeats after all 13 spokes of the equidistant pattern. All spokes are acquired exactly once. Note that a low number of 13 spokes was used for illustration only, practical RAGA trajectories would use a higher number of spokes corresponding to the Nyquist limit.

Thus, in RAGA sampling the temporal scheme is close to a golden ratio sampling scheme, but all acquired samples are members of an equidistant angular pattern. The index of the acquired spoke in the equidistant angular pattern ${ind}_{t}$ is calculated with the temporal sampling index t according to

(10) $$Z\to Z/SZ,\;t\mapsto {ind}_{t}:=\left(t\cdot G_{i-1}^{1}\right)mod\;S,$$

where Z/SZ is the additive group of integers modulo S. An illustration is provided in Figure 1. This index encodes the information about the projection angle of each acquired spoke and can be used to reorder the equidistant angular data following their temporal acquisition order.

#### 2.2.2 | Bijectivity of RAGA sampling

When acquiring spokes of an equidistant angular pattern with the increment $G_{i-1}^{1}$ following Equation (10) the mapping from t=0,⋯S-1 to Z/SZ should be bijective. If this were not the case, some spokes of the equidistant angular pattern would be sampled multiple times or not at all, which would mean that the property of the golden ratio sampling to fill each bin in an optimal way is not preserved. On the other hand, if it is bijective, then the set of S consecutive spokes of the RAGA sampling covers all positions in the equidistant scheme with S spokes. We therefore call such a bin with size S a full frame. Even with more spokes acquired, no new spoke positions will be covered. Thus, S should be chosen large enough so that the Nyquist criterion is fulfilled everywhere in k-space for a full frame, which requires $S\geq \frac{\pi }{2}m$ where m is the matrix size. Acquiring more spoke positions beyond the Nyquist limit would not provide more information even for golden ratio sampling, and a larger bin size would simply average information. This is demonstrated in Figure 6A.

To prove that we always obtain such a full frame after acquiring S spokes, we have to show that the increment $G_{i-1}^{1}$ is a generator of the cyclic additive group Z/SZ, that is, that its multiples (modulo S) generate all elements of Z/SZ. In other words, $G_{i-1}^{1}$ has order $S=G_{i}^{N}$ and this means that the pattern repeats exactly after S spokes. This is the case when the greatest common denominator of $G_{i-1}^{1}$ and $G_{i}^{N}$ is one, that is, $gcd\left(G_{i-1}^{1},G_{i}^{N}\right)=1$. The proof that the RAGA increment $G_{i-1}^{1}$ has this property can be found in Section A.

#### 2.2.3 | Approximating golden ratio angles

**FIGURE 2:**
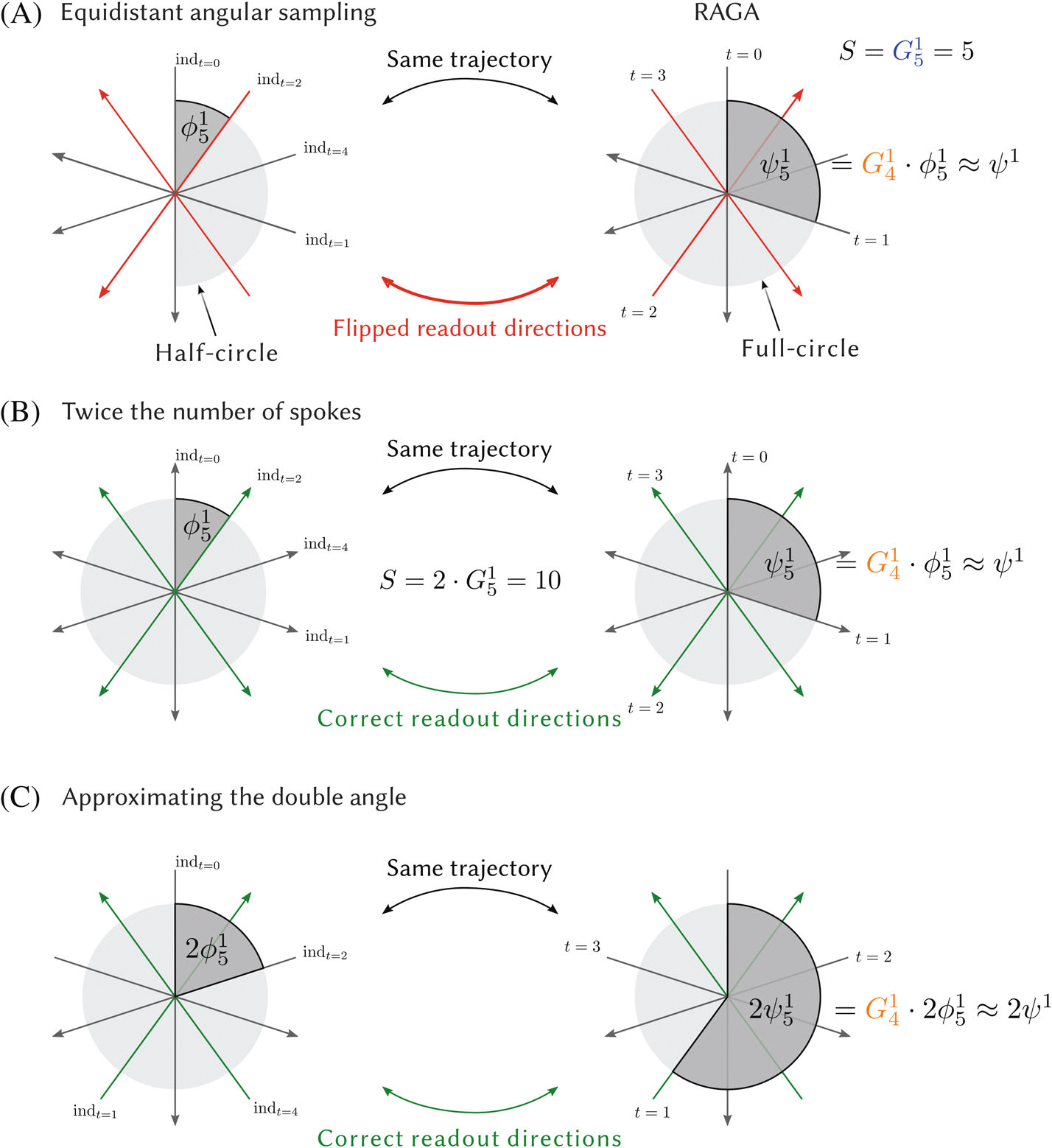
(A) An example for an equidistant angular sampling scheme with $G_{5}^{1}=5$ spokes defined over a half-circle (left) and the corresponding rational approximation of golden angles (RAGA) sampling that approximates $\psi^{1}$ with $\psi_{5}^{1}$ (right). The sampling order in RAGA corresponds to a golden ratio sampling scheme and its temporal evolution is marked with the time index t. The corresponding indices in the equidistant angular pattern ${ind}_{t}$ are calculated with the RAGA increment $G_{i-1}^{1}=G_{4}^{1}=3$ and Equation (10). Extending RAGA to the full circle leads to flipped readout directions relative to the equidistant sampling defined over a half-circle. This encoding ambiguity can be avoided by either using an extended index space or by directly covering a full circle using doubled golden ratio angles. (B) RAGA sampling using an extended space of indices. Golden ration sampling with $\psi^{1}$ is approximated with RAGA with $\psi_{5}^{1}$ by sampling a full frame with an even number $S=2G_{i}^{N}$ of spokes and using the increment $G_{i-1}^{1}$. (C) RAGA sampling approximating the doubled golden ratio angle $2\psi^{1}$ using an odd number of spokes S.

In golden ratio sampling, the sampling schemes are designed to cover a half-circle in an optimal way, because opposing spokes would not provide different information. In practice, the individual spokes are usually distributed around a full circle which improves robustness by averaging motion and other inconsistencies.^19^ Additionally, having opposing spokes can be helpful for gradient delay correction.^16,31^ If the corresponding equidistant angular pattern indexed by 0,…,S-1 is defined to sample only from one half of the circle, this leads to flipped readouts when sorting the data according to Equation (10) as illustrated in Figure 2A. This problem can be avoided by extending the space of indices to $0,\ldots ,2G_{i}^{N}-1$ to cover a full circle, reducing modulo $2G_{i}^{N}$. Spokes from the second half of the circle then correspond to an index equal or larger than $G_{i}^{N}$ (Figure 2B). Note that this does not increase the number of acquired spoke angles because the indices n and $n+G_{i}^{N}$ represent opposing spokes and this has to be taken into account when applying the Nyquist criterion. In general, when covering the full circle using S spokes, there are two different Nyquist limits for the even and the odd case:

(11) $$S\geq \left\{\begin{array}{ll} \frac{\pi }{2}m, & S\;\text{is}\;\text{odd}\\ \pi m, & S\;\text{is}\;\text{even.} \end{array}\right.$$

#### 2.2.4 | Approximating doubled golden ratio angles

Compared to sampling with an odd number of spokes, using twice as many spokes is often undesirable because this wastes space when using data files or memory buffers that use zero filling. Hence, we also consider an alternative strategy that directly covers the full circle by approximating the doubled golden ratio angle $2\psi^{N}$ using a $G_{i}^{N}$ that is odd, that is,

(12) $$2\psi^{N}\approx 2\psi_{i}^{N}=G_{i-1}^{1}\cdot 2\phi_{i}^{N}=G_{i-1}^{1}\frac{2\pi }{G_{i}^{N}}.$$

Here, the angles again correspond directly to the set of indices $0,\ldots ,G_{i}^{N}-1$ (Figure 2C). The first doubled golden ratio angles are listed in Table 2.

Thus, following Equations (7) and (10) RAGA sampling can be applied to approximate sampling with the original golden angle $\psi^{1}$, the tiny golden angles $\psi^{N}$, the doubled tiny golden angles $2\psi^{N}$, and the Golden Angle $\Phi =2\pi -2\psi^{1}$.

#### 2.2.5 | Numerical stability of RAGA sampling

In RAGA sampling, the index of the projection angle in the equidistant angular pattern follows from Equation (10) with the integers $t,\;G_{i-1}^{1}$, and S. Due to exact integer arithmetic the index ${ind}_{t}$ is bitwise reproducible. Inaccuracies due to floating point arithmetic are therefore only introduced by the multiplication of the index ${ind}_{t}$ with the base angle $\phi_{i}^{N}$ from Equation (8). This error does not propagate beyond a single repetition of the equidistant pattern rendering RAGA sampling numerically robust. In contrast, golden ratio sampling is sensitive to numerical inaccuracies which accumulate over time.

#### 2.2.6 | Data storage and processing characteristics

**FIGURE 3:**
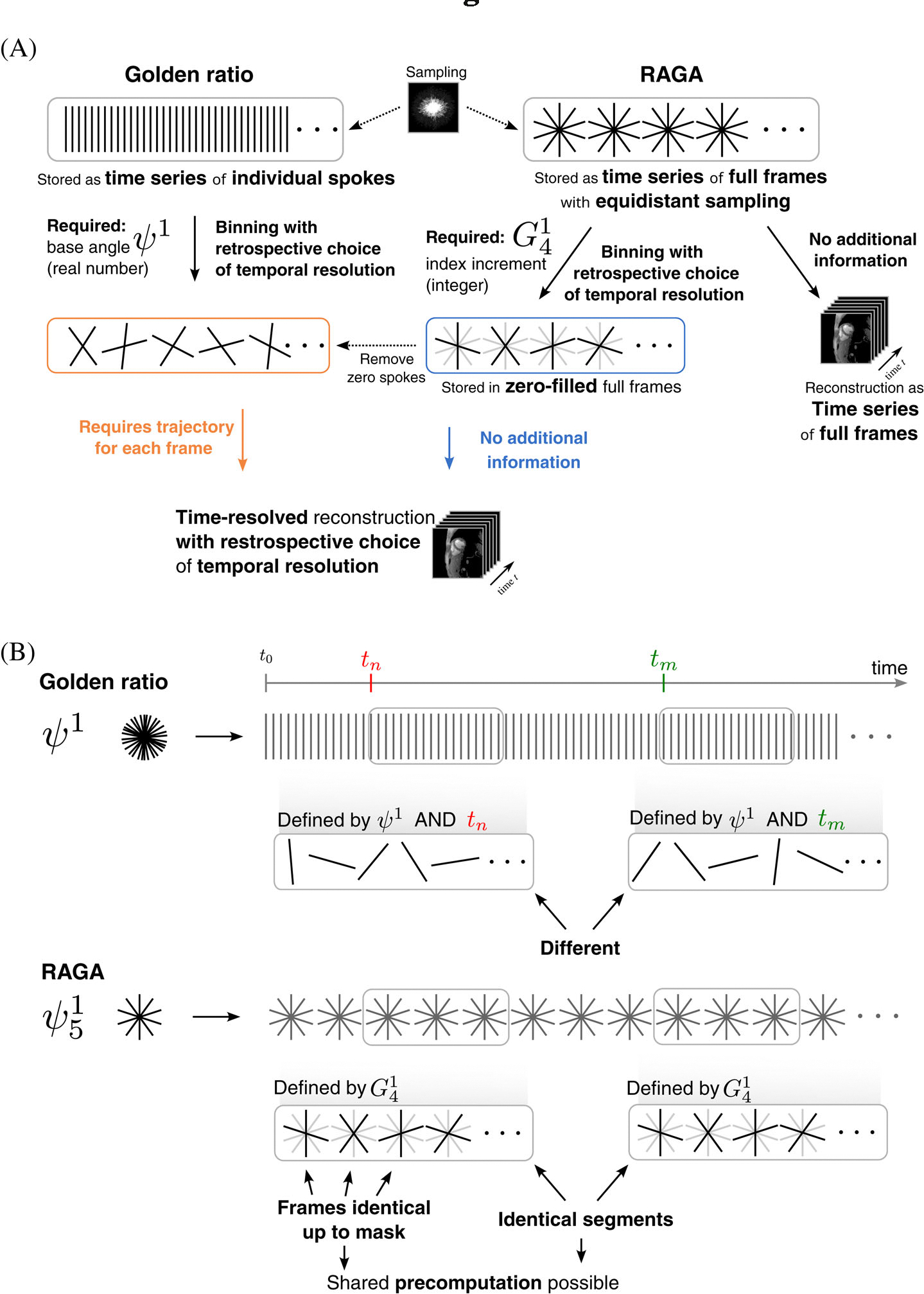
Data storage and processing for rational approximation of golden angles (RAGA) and golden ratio-based sampling. Note that the small approximation order of i=5 is chosen only for illustration purposes. (A) Golden ratio based data is stored in a time series of individual spokes and knowledge about $\psi^{1}$ is required for a reconstruction. RAGA sampling can be stored in an equidistant angular order which yields a natural decomposition of the time series into full frames. This allows reconstruction of each full frame even without any additional information. With only knowledge about the index increment $G_{4}^{1}$ corresponding to the underlying base angle (here: $\psi_{5}^{1}$), the temporal order of all spokes in the RAGA dataset can be recovered and frames with arbitrary temporal footprint can be constructed by binning as in golden ratio sampling. (B) In RAGA sampling, segments consisting of full frames can be extracted and processed without the need to keep track of their position in the original dataset. Furthermore, all full frames have identical spokes which allows sharing of precomputed data for image reconstruction. When extracting data from a nonrepeating trajectory, the position $t_{n}$ needs be known for each fragment to be able to recompute the original trajectory and no precomputed data can be shared.

In RAGA sampling all acquired samples are members of an equidistant angular pattern. This leads to the flexibility to use either of both trajectories for image reconstruction (Figure 3). With RAGA ordering, the data can be flexibly rebinned to retrospectively select the temporal resolution exactly as in golden ratio schemes. Reconstructions of golden ratio datasets require that the angle between consecutive spokes $\psi^{N}$ is stored together with the data. In contrast, for RAGA sampling the angle $\psi_{i}^{N}$ can be recovered from the total number of spokes in a full frame S and the RAGA increment $G_{i-1}^{1}$. Both are quantities which are typically stored with the data similar to the Cartesian case. A full frame of data can be compactly stored and reconstructed with a simple equidistant angular scheme. Here, not even the acquisition index of the spokes is required, and a long time series naturally decomposes into repeating full frames, which simplifies extraction and reconstruction of time periods. Given the index of the second spoke in a full frame $G_{i-1}^{1}$, the angle between temporally consecutive spokes and the full RAGA scheme can be recovered. After rebinning, the selected spokes for each frame can be stored in a zero-filled k-space corresponding to a full frame as illustrated in Figure 3.

## 3 | METHODS

### 3.1 | Implementation

To investigate the properties of the proposed sampling numerically and in MRI experiments, the RAGA sampling scheme was implemented in the Berkeley Advanced Reconstruction Toolbox.^32^ The RAGA sampling was also implemented in a radial sequence on two 3T systems using IDEA (Siemens Healthcare).

### 3.2 | Slidelobe-to-peak ratio

The properties of the PSF of the RAGA sampling schemes were evaluated by comparing the slidelobe-to-peak ratio (SPR) to golden ratio trajectories and to an equidistant angular trajectory. Trajectories consisting of 754 (for $\psi_{13}^{1}$ ), 838 (for $\psi_{10}^{7}$ ), and 419 (for $2\psi_{10}^{7}$ ) spokes were studied. With a base resolution of 200, the Nyquist criterion requires at least 314 projections.

In a sliding window approach 5–60 consecutive spokes s were extracted, combined into one frame, and the PSF for this frame was calculated by applying the adjoint nonuniform fast Fourier transformation ${\overset{ˆ}{ℱ}}^{H}$ applied to a vector of ones. From the PSF, the SPR was calculated as maximum ratio of off-center ${PSF}_{\text{off-center}}$ and center peak value ${PSF}_{\text{center}}$, that is,

(13) $$SPR=max\left(\frac{{PSF}_{\text{off-center}}}{{PSF}_{\text{center}}}\right).$$

The temporal evolution of the SPR was calculated for each window. The time series of SPR values for each window size was further processed by computing the maximum. The same analysis was performed to compare the single and double golden ratio angles: $\psi^{1},\;\psi^{7},\;2\psi^{2}$, and $2\psi^{14}$.

### 3.3 | Precomputed PSF and GROG gridding

The computational advantages when using a precomputed PSF for image reconstruction were evaluated in a simple numerical phantom experiment. Simulated datasets were created in frequency domain for the Shepp Logan phantom using a single coil, base resolution of 200, 377 radial spokes, and 100 time steps using a golden ratio angle Φ and RAGA sampling $2\psi_{12}^{2}$. The spokes were binned to 29 spokes per frame and reconstructed with an inverse nuFFT on a CPU (Intel Xeon Gold 6136 CPU @ 3.00 GHz) and GPU (Nvidia Tesla V100) using BART with and without Toeplitz embedding. For use with Toeplitz embedding when calculating the inverse nuFFT, the irrational golden ratio sampling required the calculation of a new PSF in all frames, while the selected RAGA sampling pattern repeats after 13 frames such that only 13 PSFs have to be calculated. The analysis was performed 10 times and the mean and standard derivations were calculated. Ten additional simulations with the same parameter settings but eight simulated coils were performed to compare the calibration and gridding time for GRAPPA operator gridding (GROG) preprocessing of both sampling schemes. The calibration was performed on 300 spokes and all spokes of both trajectories were gridded.

### 3.4 | Numerical stability of golden ratio trajectories

The sensitivity of golden ratio-based sampling schemes to numerical inaccuracies was investigated numerically using six different implementations. The projection angle was computed using different numerical implementations for $\psi^{1}$ and up to 500 000 repetitions corresponding to 16:40 min of continuous acquisition with a repetition time (TR) of 2 ms. Implementations using floating point arithmetic with single (**single**) and double precision (**double**) were included and compared to an implementation using quadruple precision. The computation of the projection angle over many repetitions n was studied for a multiplicative formula (·) following

(14) $$\phi_{t}=t\cdot \psi^{1}\;\text{with}\;t\in N_{0},$$

for a formula using an additive increment (+) according to

(15) $$\phi_{t}=\left\{\begin{array}{ll} \phi_{t-1}+\psi^{1} & t\in N\\ 0 & t=0 \end{array}\right.,$$

and for an additive increment followed by modular reduction after each update (+, mod) with

(16) $$\phi_{t}=\left\{\begin{array}{ll} \left(\phi_{t-1}+\psi^{1}\right)mod\;2\pi & t\in N\\ 0 & t=0 \end{array}\right..$$

RAGA angles based on $\psi_{13}^{1}$ are computed in single and double precision according to

$$\phi_{n}={ind}_{t}\cdot \frac{\pi }{G_{13}^{1}}\;\text{with}\;t\in N_{0},$$

with ${ind}_{t}$ given by Equation (10).

### 3.5 | Phantom experiment

To experimentally confirm that RAGA has similar properties to golden ratio sampling when used to retrospectively bin data, we acquired two phantom datasets. To confirm that full frames which fulfill the Nyquist criterion achieve optimal resolution, we acquired steady-state images from a static phantom with a FLASH sequence. To demonstrate that RAGA has the same properties with respect to a retrospective choice of temporal resolution, we also acquired transient magnetization using inversion-recovery (IR) FLASH.

RAGA data was acquired for a NIST (National Institute of Standards and Technology) phantom (model 106, $T_{1}$ sphere)^33^ on a Siemens Vida 3T system (Siemens Healthcare) using a 20-channel head-coil. A two-dimensional FLASH sequence (TR/TE = 3.2/2.04 ms, flip angle: 8°, bandwidth-time-product: 1.6, radiofrequency pulse duration: 0.4 ms, base resolution: 256 samples with two-fold oversampling in readout direction, field of view (FOV): 200 × 200 mm^2^) was used with RAGA sampling schemes that approximate $2\psi_{i}^{1}$ with orders i∈{7,9,10,12,13,15,16}. Additionally, data was acquired with a two-dimensional IR FLASH sequence (TR/TE = 2.9/1.77 ms, flip angle: 8°, bandwidth-time-product: 1.6, radiofrequency pulse duration: 0.4 ms, base resolution: 200 samples with two-fold oversampling in readout direction, FOV: 200×200 mm^2^) with nonselective inversion and a RAGA sampling scheme approximating $2\psi_{13}^{1}$ which corresponds to a number of spokes that fulfills the Nyquist criterion.

In a preprocessing step the gradient delays were determined with RING and a corrected trajectory was used for reconstruction.^16^ Coil profiles were calculated with ESPIRiT.^18^ The reconstruction was performed with the equidistant angular trajectory using an iterative SENSE reconstruction using the methods of conjugate gradients without regularization and a maximum number of 30 iteration steps.

The IR data was compressed from 18 coils to 12 virtual coils using a singular value decomposition.^34^ The data was retrospectively binned to 21, 55, and 233 spokes per frame. All IR datasets were reconstructed backwards in time with real-time (RT)-NLINV.^35^ The first reconstructed frames are then from the steady-state of the FLASH readouts, which is helpful when using temporal regularization. A starting regularization factor $\alpha_{0}$ of 1, a reduction factor of 1∕2 for decreasing $\alpha_{n}$ in each Gauss–Newton step, a temporal damping factor of 0.9, and 10 iterations were used for the RT-NLINV reconstruction.

### 3.6 | In vivo experiment

To investigate temporal resolution in a more complex scenario, short-axis views of a heart were acquired for a healthy volunteer in expiration after obtaining written informed consent and with approval of the local ethics board using a real-time radial two-dimensional FLASH (TR/TE = 2.9/1.77 ms, flip angle: 8°, bandwidth-time-product: 1.6, radiofrequency pulse duration: 0.4 ms, FOV: 320 × 320 mm^2^, base resolution: 200 with two-fold oversampling in readout direction) with 18 channels of a combined thorax and spine coil on a Siemens Skyra 3T system. Two datasets were acquired, one with a golden ratio angle of $\psi^{1}$ and one with the corresponding RAGA sampling using $\psi_{13}^{1}$ with 754 spokes per pattern and five repetitions.

In a preprocessing step, the data was compressed from 18 coils to 8 virtual coils using an singular value decomposition.^34^ Gradient delays were determined and the corrected trajectory is used for reconstruction. Image reconstruction was performed with RT-NLINV after binning the golden ratio and RAGA sampled datasets retrospectively to 20, 25, 30, and 40 spokes per frame. A starting regularization factor $\alpha_{0}$ of 1, a reduction factor of 1∕2, and a temporal damping of 0.9 were manually selected for the eight iterations of the RT-NLINV reconstruction. In a postprocessing step a temporal median filter was applied^35^ and the FOV was cropped to its central 150 × 150.

## 4 | RESULTS

### 4.1 | Sidelobe-to-peak ratio

**FIGURE 4:**
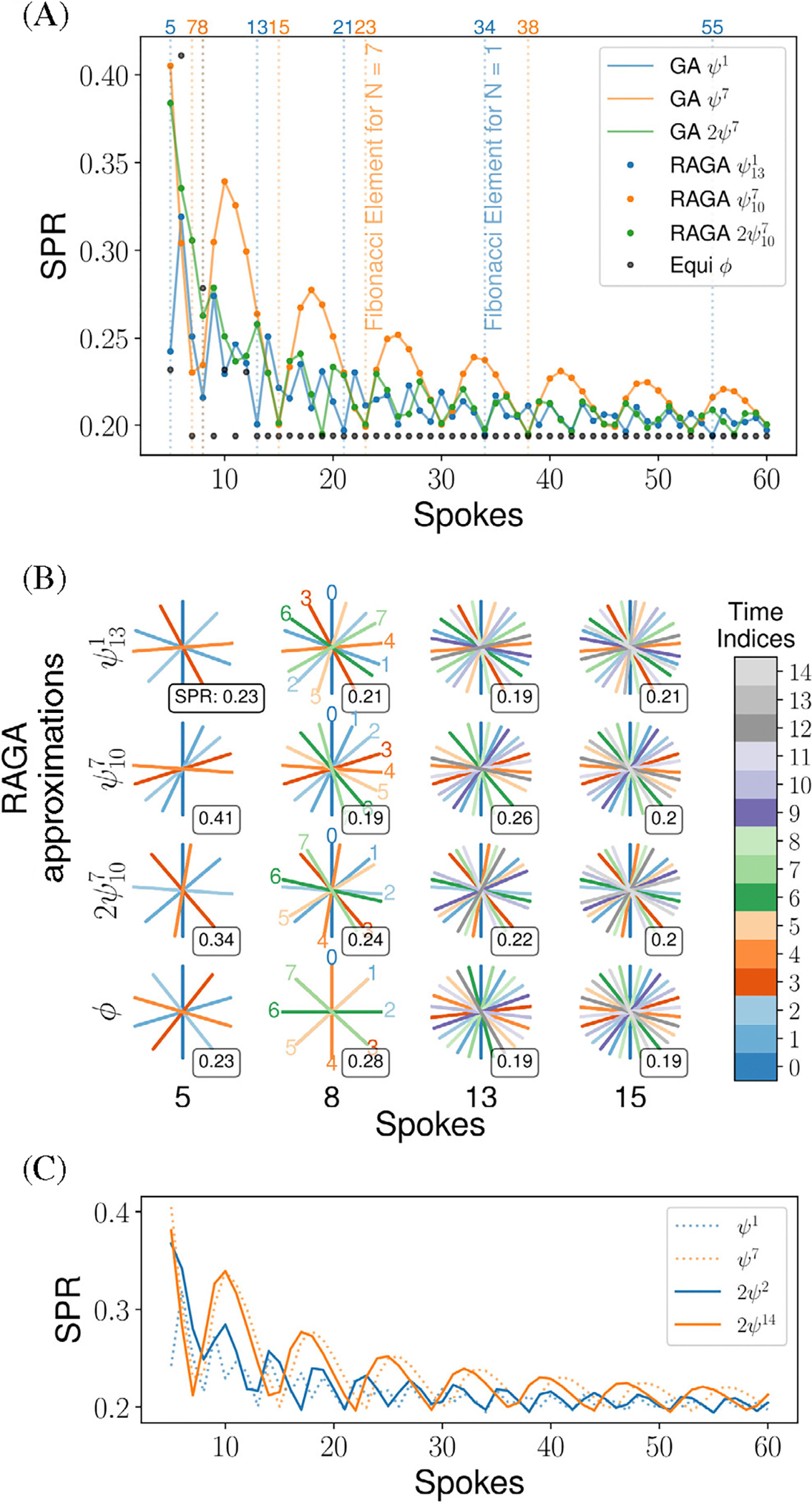
(A) Sidelobe-to-peak ratio (SPR) for sampling schemes based on the golden ratio angles $\psi^{1},\;\psi^{7}$, and $2\psi^{7}$, their rational approximations with the RAGA sampling $\psi_{13}^{1},\;\psi_{10}^{7}$, and $2\psi_{10}^{7}$, and an equidistant scheme using a base angle of ϕ. The SPR is shown for various numbers of spokes per frame. Golden ratio angles are plotted with solid lines for better differentiation from their rational approximations that are shown with colored dots. Elements of the generalized Fibonacci series are marked with dotted vertical lines. (B) Visualization of various sampling schemes for the rational approximations $\psi_{13}^{1},\;\psi_{10}^{7}$, and $2\psi_{10}^{7}$ as well as equidistant sampling with ϕ. The time index is color-coded and the calculated SPR of each sampling scheme is shown. (C) Calculated SPR for sampling schemes based on the single golden ratio angles $\psi^{1}$ and $\psi^{7}$ and similar doubled golden ratio angles $2\psi^{2}$ and $2\psi^{14}$. The SPR is shown for various numbers of spokes per frame. Single golden ratio angles are plotted with dotted lines for better differentiation from their doubled alternatives that are shown with solid lines.

Figure 4A shows the SPR values for different window sizes and sampling schemes for the angles $\psi^{1},\;\psi^{7}$, and $2\psi^{7}$ and their RAGA approximations $\psi_{13}^{1},\;\psi_{10}^{7}$, and $2\psi_{10}^{7}$. For all trajectories the SPR decreases with the number of spokes. The SPR for the equidistant angular sampling ϕ is the lowest for all trajectories except for small even numbers of spokes, which correspond to less projection angles. The trajectories based on $\psi^{7}$ and $2\psi^{7}$ share the same minima where the number of spokes corresponds to the elements of the Fibonacci series $G_{i}^{7}$, while the SPR values for the trajectory based on $\psi^{1}$ are the smallest when the number of spokes corresponds to elements of the Fibonacci series $G_{i}^{1}$. The SPR values for $\psi^{7}$ show periodically higher values compared to the angles $\psi^{1}$ and $2\psi^{7}$ as expected from their periodically changing homogeneity in k-space coverage. The single and double golden ratio angles $\psi^{1}$ and $2\psi^{7}$ have very similar SPR behavior. All RAGA approximations very closely match the results of the corresponding single and doubled golden ratio sampling schemes.

Figure 4B shows the sampling scheme using $\psi_{13}^{1},\;\psi_{10}^{7}$, and $2\psi_{10}^{7}$ for 5, 8, 13, and 15 consecutive spokes. As expected, the SPR values are large for most cases where the k-space coverage is not homogeneous. For most window sizes the equidistant angular pattern has the lowest SPR. This does not hold for small even numbers of spokes which cover fewer projection angles.

Figure 4C shows the SPR values for different window sizes and sampling schemes for the single golden ratio angles $\psi^{1},\;\psi^{7}$, and the similar double golden ratio angles $2\psi^{2}$ and $2\psi^{14}$. The same decrease in SPR for larger window sizes and the periodic SPR behavior as in Figure 4A can be observed. The single and double golden ratio angles have very similar SPR behavior which indicates that doubled golden ratio angles could be used to replace similar single golden ratio angles.

### 4.2 | Precomputed PSF and GROG gridding

**FIGURE 5:**
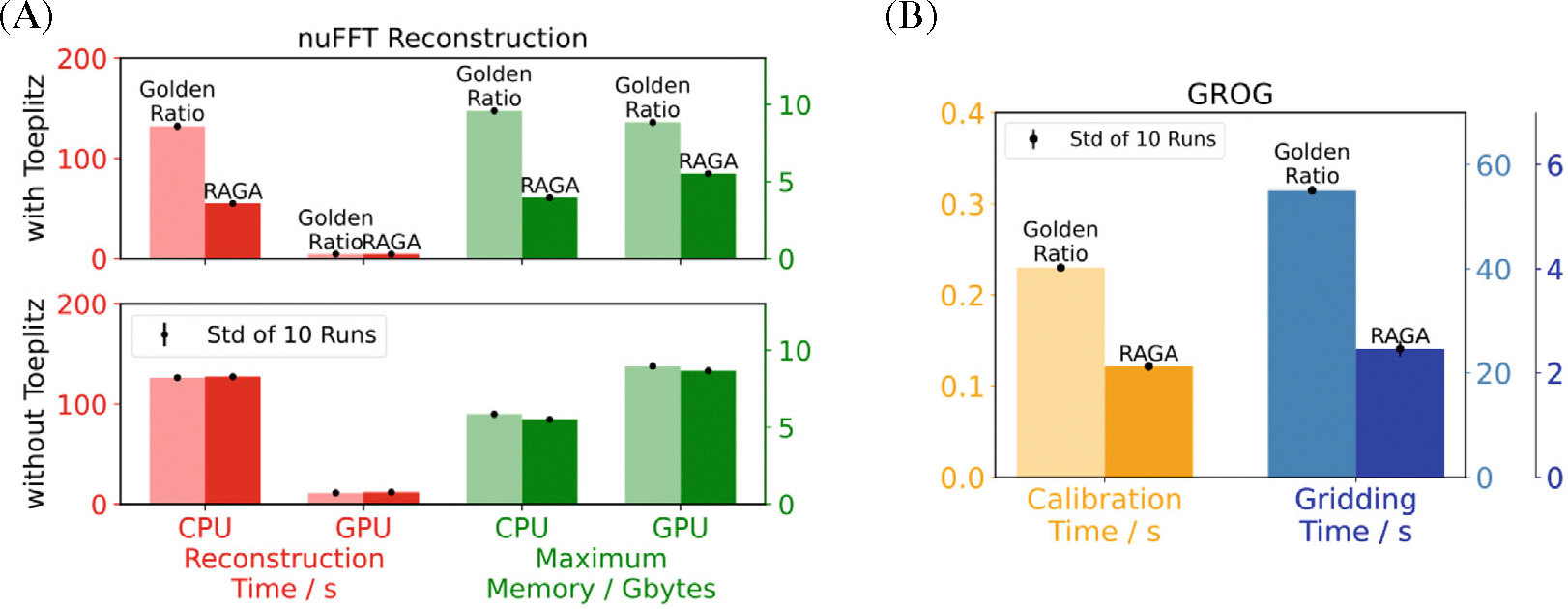
(A) Reconstruction times and memory usage of an inverse nuFFT averaged over 10 runs with and without Toeplitz embedding applied to data of the Shepp-Logan phantom simulated for trajectories with golden ratio and rational approximation of golden angles (RAGA) sampling scheme. (B) Calibration and gridding times for data preprocessing using GROG of simulated multicoil data averaged over 10 runs for golden ratio and RAGA trajectories.

Figure 5 shows the mean reconstruction times and maximum of the required memory of ten runs of an nuFFT reconstruction with and without Toeplitz embedding, GROG calibration, and GROG gridding of the Shepp-Logan phantom for golden ratio and RAGA sampling. The computational costs on both CPU and GPU are markedly higher for the reconstruction of the golden ratio sampled dataset. The GROG calibration is similarly fast for both techniques, while the gridding of the golden ratio pattern is computationally far more expensive compared to the RAGA pattern.

### 4.3 | Numerical stability of golden ratio trajectories

Table 3 shows the differences in the projection angle resulting from differing implementations of a golden ratio-based sampling scheme given by Equations (14)–(16). With the exception of the first repetition, all calculated projection angles differ from the reference due to the sensitivity of the golden ratio sampling to numerical inaccuracies. The differences resulting from floating point arithmetic with single precision are generally much higher compared to floating point operations with double precision. The multiplicative formula for the projection angle shows the lowest difference to the reference, while the additive formulas can lead to larger errors already for acquisitions times in the order of seconds when using only single precision. Avoiding the addition of numbers of different magnitude with the modular reduction after each update reduced the error markedly. For RAGA sampling, errors are on the level of the machine precision even when using single precision and do not increase over time.

### 4.4 | Phantom experiment

**FIGURE 6:**
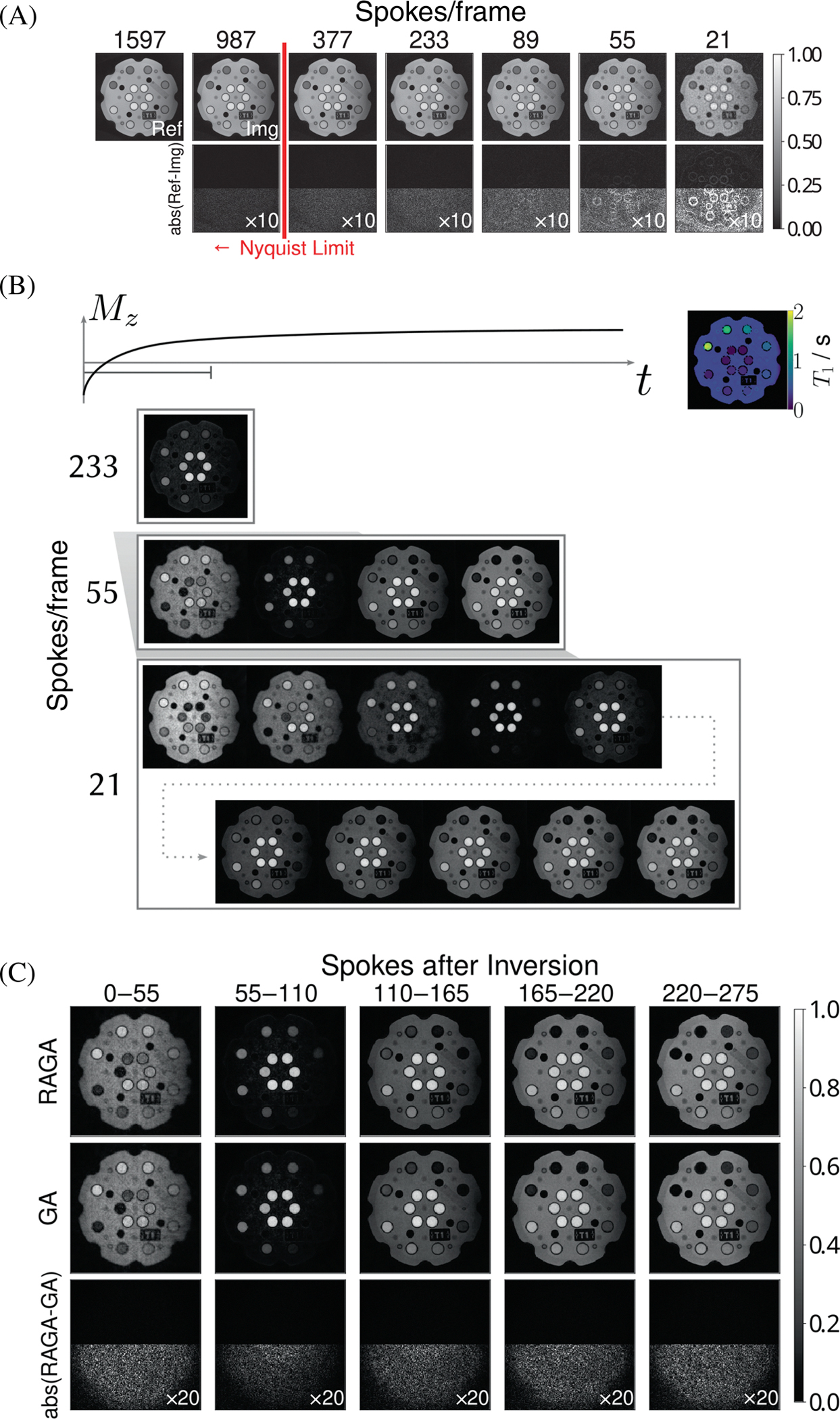
(A) Phantom images acquired with a radial FLASH sequence and reconstructed with SENSE using a different number of spokes per frame as defined by the approximation order of the rational approximation of golden angles (RAGA) sampling for $2\psi_{i}^{1}$ with orders i∈{7,9,10,12,13,15,16}. Difference maps to the frame with the most spokes are shown, with the bottom half scaled by 10. (B) Visualization of a series of phantom images acquired with an inversion-recovery (IR) FLASH sequence and RAGA sampling with spoke angle $2\psi_{13}^{1}$. The data was rebinned to 21, 55, and 233 spokes per frame and reconstructed using RT-NLINV demonstrating the increasing temporal resolution. A T_1_ map was calculated from the rebinned dataset with the highest temporal resolution, that is, 21 spokes per frame. (C) Selected frames from the time series showing the inversion-recovery acquired with 55 spokes per frame using RAGA angle $2\psi_{13}^{1}$. The difference maps show the difference to an acquisition with golden ratio angle $2\psi^{1}$ with the bottom half scaled up by 20.

Figure 6A shows different reconstructions of a FLASH sequence acquired with RAGA sampling schemes of varying approximation order. This results in different numbers of spokes per frame. The differences of the individual maps with respect to the map with the most acquired spokes are shown. The error increases slightly when the number of spokes is reduced, but does not include any structural details until fewer than 89 spokes per frame are used. The Nyquist limit in this experiment corresponds to 402 spokes per frame. From this point on, increasing the number of projection angles does not add more information.

The rows in Figure 6B show differently binned datasets with varying temporal resolution covering almost the same time interval during the IR FLASH experiment. With a reduced number of spokes per frame more states during the recovery of the magnetization can be resolved.

Figure 6C shows reconstructions of two IR FLASH experiments acquired with a golden ratio and RAGA sampling scheme. Their differences are low in each time interval during the recovery and appear noise-like without structural details. This confirms that both schemes have the same flexibility in choosing the temporal resolution retrospectively.

### 4.5 | In vivo experiment

**FIGURE 7:**
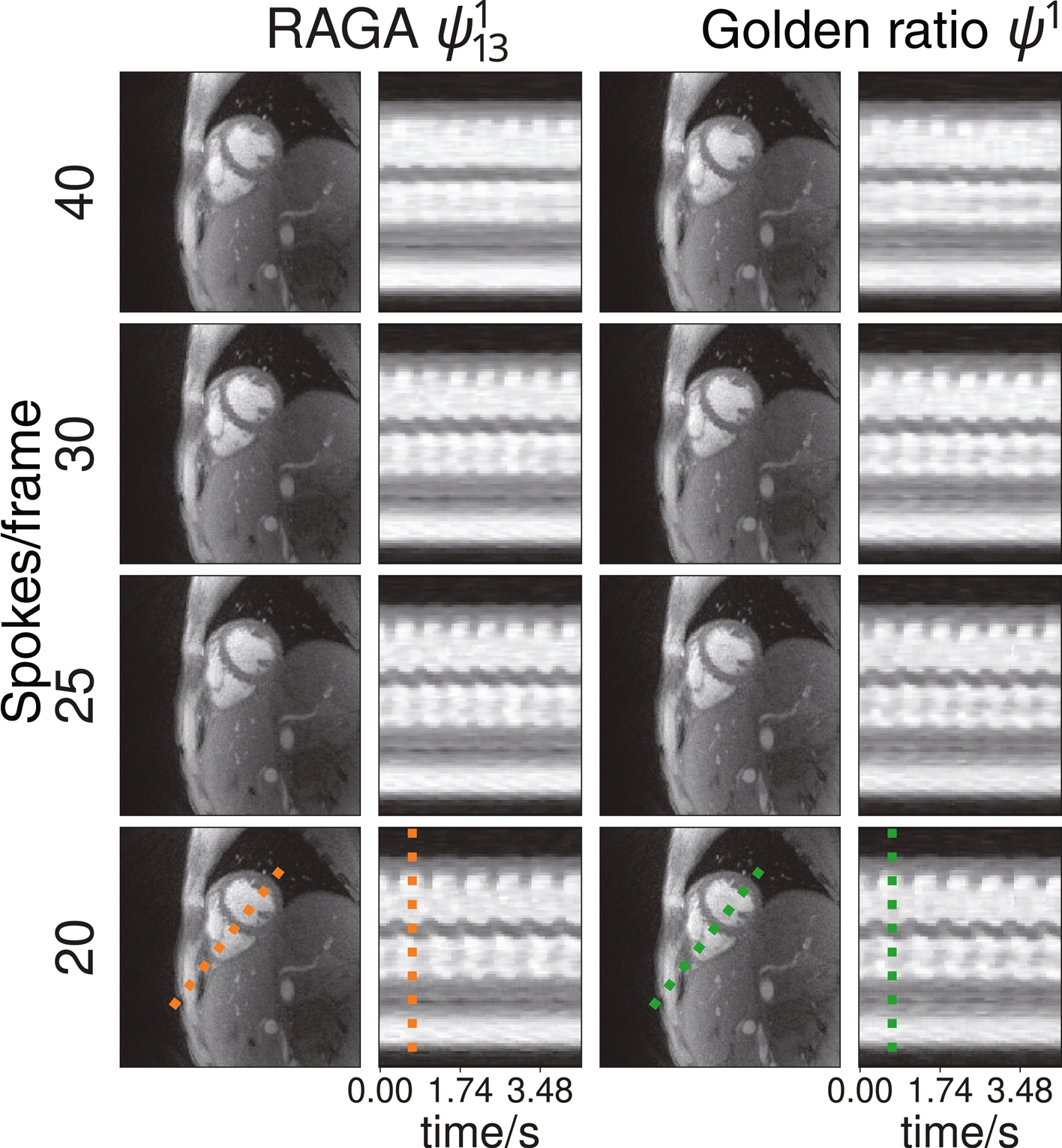
Short-axis views of a human heart acquired with a radial FLASH sequence using rational approximation of golden angles (RAGA) (full frame of 233 spokes) and golden ratio sampling. Both datasets were reconstructed using RT-NLINV after retrospective binning to different temporal resolutions. The left two rows show an image from the diastolic phase sampled with the rational approximation $\psi_{13}^{1}$ and the time evolution of an extracted line profile. The right shows the same analysis for the golden ratio angle $\psi^{1}$.

Figure 7 shows the RT-NLINV reconstructions of a real-time FLASH acquisition of a human heart in short-axis view for different windows sizes for RAGA and golden ratio sampling. A small difference in slice localization can be observed between both acquisition with $\psi_{13}^{1}$ and $\psi^{1}$ likely caused by motion or breathing. The reconstruction quality of the acquisition with RAGA approximation $\psi_{13}^{1}$ and with the irrational angle $\psi^{1}$ is visually very similar to that of golden ratio sampling. The extracted line profiles that show a temporal profile of the cardiac motion are also very similar for all temporal resolutions even for time intervals with fast cardiac motion. In summary, the golden ratio and RAGA sampling provide essentially the same image quality over multiple heart cycles and temporal resolutions.

## 5 | DISCUSSION

Golden ratio-based sampling schemes are well suited for dynamic MRI because they allow a retrospective choice of temporal resolution. This work simplifies the concept of golden ratio sampling by reducing them to a reordering scheme for the simpler equidistant sampling strategy. This enables the sharing of precomputed information in reconstruction algorithms, improves numerical robustness, and simplifies data management. A crucial insight was that with the appropriate choice of the spoke increment and total number of spokes, the RAGA sampling scheme not only approximates the right angle but also steps through all possible angles of the underlying equidistant sampling scheme.

Although based on a rational approximation, we show that RAGA sampling preserves all important properties of golden ratio sampling. This was demonstrated by calculating and comparing the SPR values of selected irrational angles with their RAGA approximations. For spokes following the corresponding generalized Fibonacci series the SPR values of both schemes are close to the theoretical optimum of the equidistant angular distribution. For spokes in between elements of the generalized Fibonacci series the periodically changing homogeneity in k-space coverage for small angles leads in both schemes to increased SPR values. When using retrospective binning based on a motion signal, individual bins can also have incomplete k-space coverage and increased SPR for both golden ratio and RAGA sampling. This is more likely when using RAGA sampling with insufficiently low approximation order. In general in RAGA sampling, the approximation accuracy increases fast with the approximation order. For sampling schemes that fulfill the Nyquist criterion for typical base resolutions, the approximation error is lower than one degree.

Because golden ratio sampling is based on a definition using a half circle, while its practical application then typically uses angles from a full circle, a one-to-one application of RAGA leads to flipped readouts. This can be avoided by either using doubled golden ratio angles as base angles or by using a doubled index space. Doubled golden ratio angles have very similar SPR behavior to similar golden ratio angles and provide an alternative that can be used to distribute the spokes over the full circle without having to artificially increase the index space.

In contrast to golden ratio sampling schemes, RAGA sampling schemes repeat after acquiring a finite number of spokes as defined by the approximation order. This avoids the accumulation of numerical errors over multiple repetitions due to floating point arithmetic or differing numerical implementations. Single precision arithmetic has to be avoided for golden ratio sampling due to high numerical errors. While double precision can be very accurate even for longer scan times and numerical accuracy is then not a limitation, one has to avoid numerically poor implementations and agree on a single numerical implementation for exact reproduction. In contrast, RAGA trajectories are numerically robust for arbitrarily long scans even when using single precision. Additionally, the sampling scheme and related quantities such as its PSF can be precomputed and reused in Toeplitz-accelerated non-Cartesian reconstructions, reducing memory requirements and reconstruction time. Methods for accelerating non-Cartesian reconstructions with preprocessing based on GROG benefit from reduced gridding costs, because the individual shifting kernels $G_{x}^{\delta x}$ and $G_{y}^{\delta y}$ can be reused for samples on repeating trajectories.

Golden ratio sampling acquires new projections of the object with every spoke for the complete time series. While this seems to be an advantage, once the Nyquist criterion is exceeded further projection angles do not add additional information compared to their repeated variants in RAGA sampling. This was confirmed in a phantom experiment by using a varying number of spokes per frame. Their comparison showed that structural differences in the reconstructions only appeared below the Nyquist limit. This can be used in RAGA sampling to automatically determine the required approximation order by using the Nyquist criterion to determine the maximum number of useful projection angles, further removing an unnecessary degree of freedom for the user. The sampling scheme is then fully defined by the approximated angle $\psi^{N}$ and the base resolution, while the order i is automatically set to the lowest value, which still fulfills the Nyquist criterion.

Compared to golden ratio sampling, RAGA sampling comes with the flexibility to use either an equidistant angular or retrospectively rebinned temporal trajectory for image reconstruction as demonstrated for IR-FLASH and a real-time cardiac MRI data set. The data can be conveniently stored and reconstructed without any additional information as known from Cartesian data. This can be expected to become even more useful when sampling schemes are more complicated with additional encoding dimensions that then often combine different sampling strategies. In addition, navigator lines are often repeated exactly and can simply be reused in the reconstruction by inserting them into the corresponding integer position, while this would require special handling in conventional golden ratio sampling. Finally, repeated patterns enable precomputation of interpolation weights, PSF, subspace coefficients, or other quantities that can be shared at the level of full frames or individual spokes. As shown in this work, this can reduce computational demand substantially. RAGA data is almost self-explanatory and simplifies data management in various ways.

In the future, similar schemes could be developed for three-dimensional radial trajectories.^36,37^

## 6 | CONCLUSION

In this work, we introduced RAGA sampling schemes as simplified versions of golden ratio sampling using rational approximations. We proved mathematically that this leads to temporal sampling schemes which correspond to equidistant sampling after reordering, which allows precomputation in reconstruction algorithms, improves numerical robustness, and simplifies data management. At the same time, the approximations preserve all the practical advantages of golden ration sampling, which was shown using a numerical analysis and phantom and in vivo experiments.

## Supplementary Material

Supp1 **Video S1.** Video of short-axis views of a human heart acquired with real-time radial FLASH using RAGA (full frame of 233 spokes) and golden ratio sampling. Both datasets were reconstructed using RT-NLINV after retrospective binning to different temporal resolutions. The left shows the reconstructed dataset sampled with the rational approximation $\psi_{13}^{1}$ and the right shows the reconstructions of the data sampled with the golden ratio angle $\psi^{1}$.

## Figures and Tables

**Table 1:** Rational approximation of various golden ratio-based angles $\psi^{N}$ for different approximation orders i.

Approximated golden ratio angles									
			$\psi^{N=1}$	$\psi^{2}$	$\psi^{3}$	$\psi^{4}$	$\psi^{5}$	$\psi^{6}$	$\psi^{7}$
Approximation order i	2	$\psi_{2}^{N}$	180.000°	90.000°	60.000°	45.000°	36.000°	30.000°	25.714°
		$G_{1}^{1}/G_{2}^{N}$	1/1	1/2	1/3	1/4	1/5	1/6	1/7
	3	$\psi_{3}^{N}$	90.000°	60.000°	45.000°	36.000°	30.000°	25.714°	22.500°
		$G_{2}^{1}/G_{3}^{N}$	1/2	1/3	1/4	1/5	1/6	1/7	1/8
	4	$\psi_{4}^{N}$	120.000°	72.000°	51.429°	40.000°	32.727°	27.692°	24.000°
		$G_{3}^{1}/G_{4}^{N}$	2/3	2/5	2/7	2/9	2/11	2/13	2/15
	5	$\psi_{5}^{N}$	108.000°	67.500°	49.091°	38.571°	31.765°	27.000°	23.478°
		$G_{4}^{1}/G_{5}^{N}$	3/5	3/8	3/11	3/14	3/17	3/20	3/23
	6	$\psi_{6}^{N}$	112.500°	69.231°	50.000°	39.130°	32.143°	27.273°	23.684°
		$G_{5}^{1}/G_{6}^{N}$	5/8	5/13	5/18	5/23	5/28	5/33	5/38
	7	$\psi_{7}^{N}$	110.769°	68.571°	49.655°	38.919°	32.000°	27.170°	23.607°
		$G_{6}^{1}/G_{7}^{N}$	8/13	8/21	8/29	8/37	8/45	8/53	8/61
	8	$\psi_{8}^{N}$	111.429°	68.824°	49.787°	39.000°	32.055°	27.209°	23.636°
		$G_{7}^{1}/G_{8}^{N}$	13/21	13/34	13/47	13/60	13 / 73	13 / 86	13 / 99
	9	$\psi_{9}^{N}$	111.176°	68.727°	49.737°	38.969°	32.034°	27.194°	23.625°
		$G_{8}^{1}/G_{9}^{N}$	21/34	21/55	21/76	21/97	21/118	21/139	21/160
	10	$\psi_{10}^{N}$	111.273°	68.764°	49.756°	38.981°	32.042°	27.200°	23.629°
		$G_{9}^{1}/G_{10}^{N}$	34/55	34/89	34 / 123	34/157	34 / 191	34/225	34/259
	11	$\psi_{11}^{N}$	111.236°	68.750°	49.749°	38.976°	32.039°	27.198°	**23.628°**
		$G_{10}^{1}/G_{11}^{N}$	55/89	55/144	55/199	55/254	55/309	55/364	55/419
	12	$\psi_{12}^{N}$	111.250°	68.755°	49.752°	**38.978°**	32.040°	**27.199°**	**23.628°**
		$G_{11}^{1}/G_{12}^{N}$	89 / 144	89/233	89/322	89/411	89 / 500	89 / 589	89 / 678
	13	$\psi_{13}^{N}$	111.245°	**68.753°**	**49.750°**	**38.977°**	**32.040°**	**27.198°**	**23.628°**
		$G_{12}^{1}/G_{13}^{N}$	144 / 233	144/377	144 / 521	144 / 665	144 / 809	144/953	144/1097
	14	$\psi_{14}^{N}$	**111.247°**	68.754°	**49.751°**	**38.978°**	**32.040°**	**27.198°**	**23.628°**
		$G_{13}^{1}/G_{14}^{N}$	233/377	233/610	233/843	233 / 1076	233 / 1309	233/1542	233 / 1775

*Notes*: Each approximation $\psi_{i}^{N}$ is shown with their corresponding fraction using generalized Fibonacci numbers. The bold numbers on dark gray shaded background mark sampling schemes that fulfill the Nyquist criterion for a base resolution of 200, that is, contain more than 314 projection angles. The rows containing angles are highlighted with a light gray shading for an improved differentiation from their corresponding Fibonacci number ratios.

**Table 2:** Table listing the first seven single golden ratio angles and the first 14 doubled golden ratio angles.

Golden ratio angles (°)						
$\psi^{1}$	$\psi^{2}$	$\psi^{3}$	$\psi^{4}$	$\psi^{5}$	$\psi^{6}$	$\psi^{7}$
111.246	68.754	49.751	38.978	32.040	27.198	23.628
Doubled golden ratio angles (°)						
$2\psi^{1}$	$2\psi^{2}$	$2\psi^{3}$	$2\psi^{4}$	$2\psi^{5}$	$2\psi^{6}$	$2\psi^{7}$
222.492	137.508	99.502	77.955	64.079	54.397	47.256
$2\psi^{8}$	$2\psi^{9}$	$2\psi^{10}$	$2\psi^{11}$	$2\psi^{12}$	$2\psi^{13}$	$2\psi^{14}$
41.773	37.430	33.905	30.986	28.531	26.436	24.627

**Table 3:** The projection angles for selected repetitions of a continuous golden ratio-based sampling scheme with base angle $\psi^{1}$ and for the RAGA approximation $\psi_{13}^{1}$.

		Repetitions				
		10 000	25 000	100 000	250 000	500 000
	Golden Ratio Ref (°)	61.18	152.95	251.80	89.49	178.99
	RAGA Ref (°)	46.35	115.88	103.52	78.80	157.60
Golden ratio	single_(⋅)_ (°)	−2.3e−02	−1.4e−03	−5.7e−03	−4.6e−01	−9.2e−01
	single_(+)_ (°)	−2.7e+02	−9.5e+01	1.7e+02	−1.4e+02	7.0e+01
	single_(+,mod)_ (°)	−1.1e−01	−2.7e−01	−1.1e+00	−2.7e+00	−5.4e+00
	single_RAGA_ (°)	−3.4e−06	−9.7e−11	−3.6e−06	−4.1e−06	−8.2e−06
Golden ratio	double_(⋅)_ (°)	1.3e−10	2.2e−10	8.6e−10	4.9e−10	9.8e−10
	double_(+)_ (°)	2.5e−07	9.4e−07	4.4e−06	−1.8e−04	−5.9e−04
	double_(+,mod)_ (°)	7.2e−11	1.8e−10	7.2e−10	1.8e−09	3.6e−09
	double_RAGA_ (°)	−3.1e−15	−1.4e−15	−5.7e−15	−1.5e−15	−2.9e−15
	Acquisition (min:s)	0:20	0:50	3:20	8:20	16:40

*Notes*: The acquisition times are computed assuming TR = 2 ms. Different implementations of the calculation of the projection angle are compared. **Single** and **double** refer to the floating point precision as single or double, respectively, and (⋅), (+), and (+, mod) refer to the calculation of the projection angle in time according to Equations (14)–(16). Angles and errors were converted to degrees for representation. Machine epsilons scaled in the same way are 6.8 × 10^−6^ and 1.3 × 10^−14^ for single and double precision, respectively. The gray shading of individual rows is added to improve the visual differentiation between the reference angles and the calculation differences for golden ratio as well as RAGA sampling.

## Data Availability

The tools of this work are implemented in the Berkeley Advanced Reconstruction Toolbox (Github:mrirecon/bart, DOI:10.5281/zenodo.592960) with commit *79fd4a72*. They will be part of the future release following version 0.9.00 of the software. The scripts to reproduce all the figures and tables can be found at Github:mrirecon/raga (DOI:10.5281/zenodo.11287833). The required datasets can be downloaded from Zenodo (DOI: 10.5281/zenodo.10260250). An interactive tutorial demonstrating the various storage option accessible with RAGA sampling and illustrated in Figure 3 can be found on Github:mrirecon/raga-tutorial (DOI:10.5281/zenodo.13333245).

## APPENDIX A. BIJECTIVITY OF RAGA SAMPLING

### Theorem 1.

For all i,N∈N with i>1 the mapping $t\mapsto {ind}_{t}:=t\cdot G_{i-1}^{1}mod\;G_{i}^{N}$ bijectively allocates all indices $\left[0,G_{i}^{N}-1\right]$.

The proof requires two lemmata:

### Lemma 1.

For i>2, the generalized Fibonacci series can also be expressed as

(A1)
$$G_{i}^{N}=NG_{i-1}^{1}+G_{i-2}^{1}.$$

#### Proof.

The assertion is shown via induction over i. From the definition of the generalized Fibonacci sequence Equation (5) the assertion follows for i=3 and i=4:

(A2)
$$\begin{array}{l} G_{3}^{N}=G_{2}^{N}+G_{1}^{N}=N+1=NG_{2}^{1}+G_{1}^{1} \end{array}\;and$$

(A3)
$$\begin{array}{l} G_{4}^{N}=G_{3}^{N}+G_{2}^{N}=N+1+N\\ \;=N\cdot 2+1=NG_{3}^{1}+G_{2}^{1}. \end{array}$$

Assuming the assertion (A1) for i-1 and i and applying it to both terms on the right side of the definition (5) of the generalized Fibonacci sequence yields

$$\begin{array}{l} G_{i+1}^{N}\;=G_{i}^{N}+G_{i-1}^{N}\\ \;=\left(NG_{i-1}^{1}+G_{i-2}^{1}\right)+\left(NG_{i-2}^{1}+G_{i-3}^{1}\right)\\ \;=N\left(G_{i-1}^{1}+G_{i-2}^{1}\right)+\left(G_{i-2}^{1}+G_{i-3}^{1}\right)=NG_{i}^{1}+G_{i-1}^{1}, \end{array}$$

where the last step uses the definition of the standard Fibonacci sequence. ▪

### Lemma 2.

For $i\in N,\;i>1,\;gcd\left(G_{i-1}^{1},G_{i}^{N}\right)=1$.

#### Proof.

For i=2, we can compute $gcd\left(G_{1}^{1},G_{2}^{N}\right)=1$. For i>2, we first prove the assertion for N=1 by induction over i. With the definition of the Fibonacci sequence and the properties of the greatest common divisor, we obtain

$$\begin{array}{l} gcd\left(G_{i-1}^{1},G_{i}^{1}\right)\;=gcd\left(G_{i-1}^{1},G_{i-1}^{1}+G_{i-2}^{1}\right)\\ \;=gcd\left(G_{i-1}^{1},G_{i-2}^{1}\right). \end{array}$$

Hence, the assertion also follows for all i>2 by induction. Using Lemma 1 we can extend these results to N>1 by computing

$$\begin{array}{l} gcd\left(G_{i-1}^{1},G_{i}^{N}\right)=gcd\left(G_{i-1}^{1},NG_{i-1}^{1}+G_{i-2}^{1}\right)\\ \;=gcd\left(G_{i-1}^{1},G_{i-2}^{1}\right)=1. \end{array}$$

#### Proof of Theorem 1.

We prove injectivity by contradiction: Assuming there exist $k,l\in \left[0,G_{i}^{N}-1\right]$ with k≠l that are mapped to the same residue class, that is,

$$k\cdot G_{i-1}^{1}\equiv l\cdot G_{i-1}^{1}mod\;G_{i}^{N}.$$

From Lemma 2 we have that $G_{i-1}^{1}$ and $G_{i}^{N}$ are coprime, which then implies $k\equiv l\;mod\;G_{i}^{N}$ by the rules of modular arithmetic. But this contradicts the assumption and therefore proves injectivity. Surjectivity follows from injectivity by observing that the cardinality of the domain and the cardinality of the codomain of the mapping are the same and finite. ■
